# Ventromedial prefrontal cortex/anterior cingulate cortex Glx, glutamate, and GABA levels in medication-free major depressive disorder

**DOI:** 10.1038/s41398-021-01541-1

**Published:** 2021-08-05

**Authors:** Joshua T. Kantrowitz, Zhengchao Dong, Matthew S. Milak, Rain Rashid, Lawrence S. Kegeles, Daniel C. Javitt, Jeffrey A. Lieberman, J. John Mann

**Affiliations:** 1 Department of Psychiatry, Columbia University, College of Physicians and Surgeons, New York, NY USA; 2 New York State Psychiatric Institute, New York, NY USA; 3 Nathan Kline Institute, 140 Old Orangeburg Road, Orangeburg, NY USA; 4 Department of Radiology, Columbia University, College of Physicians and Surgeons, New York, NY USA

**Keywords:** Physiology, Diagnostic markers, Depression, Predictive markers

## Abstract

Glutamate (Glu) and gamma-aminobutyric acid (GABA) are implicated in the pathophysiology of major depressive disorder (MDD). GABA levels or GABAergic interneuron numbers are generally low in MDD, potentially disinhibiting Glu release. It is unclear whether Glu release or turnover is increased in depression. Conversely, a meta-analysis of prefrontal proton magnetic resonance spectroscopy (^1^H MRS) studies in MDD finds low Glx (combination of glutamate and glutamine) in medicated MDD. We hypothesize that elevated Glx or Glu may be a marker of more severe, untreated MDD. We examined ventromedial prefrontal cortex/anterior cingulate cortex (vmPFC/ACC) Glx and glutamate levels using ^1^H MRS in 34 medication-free, symptomatic, chronically ill MDD patients and 32 healthy volunteers, and GABA levels in a subsample. Elevated Glx and Glu were observed in MDD compared with healthy volunteers, with the highest levels seen in males with MDD. vmPFC/ACC GABA was low in MDD. Higher Glx levels correlated with more severe depression and lower GABA. MDD severity and diagnosis were both linked to higher Glx in vmPFC/ACC. Low GABA in a subset of these patients is consistent with our hypothesized model of low GABA leading to glutamate disinhibition in MDD. This finding and model are consistent with our previously reported findings that the NMDAR-antagonist antidepressant effect is proportional to the reduction of vmPFC/ACC Glx or Glu levels.

## Introduction

Major depressive disorder (MDD) is a leading cause of disability worldwide. Convergent evidence from in vivo brain imaging, postmortem brain and gene expression studies suggests that dysfunction of both glutamatergic and gamma-aminobutyric acid (GABAergic) systems play a role in the pathophysiology of both MDD and bipolar depression (BPD) [1–7]. Glutamate is the most abundant excitatory neurotransmitter in the brain and the endogenous ligand for *N*-methyl-d-aspartate-type glutamate receptor (NMDAR) and α-amino-3-hydroxy-5-methylisoxazole-4-propionic acid receptors (AMPAR) [8]. GABA is the most abundant inhibitory neurotransmitter. These findings suggest a hypothetical model of mood disorders whereby deficient GABAergic inhibition of the cortical glutamatergic system results in its overactivity.

This model is supported by treatment studies showing that NMDAR antagonists, such as ketamine and d-cycloserine (DCS), have antidepressant benefit in both MDD [9–11] and BPD [12, 13]. Moreover, some GABA agonists show promise as antidepressants [14]. Better understanding of glutamatergic and GABA pathophysiology in depression may aid identification of new effective antidepressant medications, that can act rapidly like ketamine, but can be used orally and have fewer side effects and less abuse potential than ketamine [15, 16].

Proton magnetic resonance spectroscopy (^1^H MRS) is a noninvasive way to measure regional concentrations of brain metabolites, including glutamate, Glx (combined glutamate + glutamine), and GABA. In our recent work, we have used ^1^H MRS to quantify NMDAR-antagonist effects on these neurotransmitters in both healthy [17, 18] and depressed [19–21] individuals. We utilized a voxel that included ventromedial prefrontal cortex and adjacent anterior cingulate cortex (vmPFC/ACC), because of evidence implicating these regions in the pathogenesis of mood disorders [22–24], and microdialysis rodent [25, 26] and clinical studies of MDD finding a glutamatergic surge centered in vmPFC/ACC in response to NMDAR antagonists [19, 20].

Further support for glutamatergic pathophysiology in this area comes from a ^1^H MRS meta-analysis of 25 studies of the medial prefrontal cortex (mPFC) [27] where a subgroup analysis found Glx levels in mPFC were lower in medicated (SMD = −0.5, *P* = 0.001), but not in medication-free (SMD = −0.27, *P* = 0.27) MDD vs. HV. Although the meta-analysis ROI does not precisely overlap our ROI, this meta-analysis’s findings are consistent with our results [20, 21] showing that improvement in depression in response to NMDAR-antagonist administration correlated with lower Glx or Glu in vmPFC/ACC.

Although there is disagreement, several approaches also suggest there is a GABAergic deficiency in mood disorders. A 2016 meta-analysis found GABA deficits in actively depressed (SMD = −0.52, *P* = 0.005), but not in remitted patients (SMD = −0.24, *P* = 0.31) across all brain regions in MDD [28]. More recent studies of prefrontal GABA levels in depression have been inconsistent [29, 30]. Postmortem studies of GABA neurons find deficits in mood disorders [31–33]. CSF GABA studies also do not show consistent results, but we have found that CSF GABA declines with age and lower CSF GABA correlates with more severe depression-associated anxiety in unmedicated patients [7].

These findings indicate a model of mood disorders that involve impaired GABAergic inhibition of glutamatergic activity. We proposed to test this model using ^1^H MRS measurement of glutamate, Glx, and GABA in medication-free MDD. Recent meta-analyses of such studies are notable for the inclusion of mostly medicated subjects, examination of brain regions less clearly associated with mood disorders such as occipital cortex, measurement of Glx alone as opposed to concurrent measurement of glutamate and GABA, and the small sample sizes of individual studies (e.g., mean 22 MDD and 20 HV in [27]). In our present study, we focused on vmPFC/ACC Glx, glutamate, and GABA in a comparatively large group of MDD and HV. Our hypothesized model predicted that we would find high Glu and Glx, and low GABA in recently unmedicated MDD.

## Methods

### Participants

All subjects signed informed consent, and all studies were approved by the New York State Psychiatric Institute IRB.

As previously reported [20], 34 physically healthy patients meeting DSM-IV criteria for a major depressive episode (MDE) in the context of MDD participated, as part of a dose-finding trial of ketamine. At screening, MDD patients were actively depressed, with a Montgomery–Åsberg Depression Rating Scale (MADRS) total score ≥22 and either currently unmedicated or judged to be clinically safe for a washout of currently ineffective medications under the supervision of the study psychiatrist. No patients were taken off medications that were working effectively for the purpose of enrolment in this study. Patients were medication-free prior to MRI scanning for at least 14 days, and off neuroleptics for at least 1 month, and off fluoxetine for at least 6 weeks before scanning.

As previously reported [17, 18], HVs were physically healthy men and women without current or past Axis I or II psychiatric or substance use disorder history, and unmedicated when scanned.

For all participants, baseline, e.g., pre-NMDAR-antagonist, ^1^H MRS measurements were used.

### MRI/MRS data acquisition and processing

Details of MRI/^1^H MRS data acquisition and processing are given in Supplementary Materials and are summarized here. All ^1^H MRS data were acquired on a General Electric SIGNA Premier 3 T MR scanner employing a 3.0 × 2.5 × 2.5 cm^3^ voxel located in the vmPFC/ACC with the center of the posterior side of the voxel close to the genu of the corpus callosum (Supplementary Fig. 1). The ^1^H MRS sequences were PROBE-J for 34 MDD and 12 HVs and PROBE-P for another 20 HVs [17, 18, 20]. All data were subjected to the same preprocessing steps, and partial volume correction and relaxation correction were performed on both water and metabolites signals for quantification of metabolites (Supplementary Tables 1 and 2). Since two ^1^H MRS sequences were used, we used phantoms quantified using the two sets of parameters to rule out any meaningful effect on results (see details in Supplemental Materials). The spectral quality of both datasets was good or acceptable. We did not observe differences in spectral quality, and we did not exclude any data from any groups (see details in Supplementary Materials).

We used NAA as a normalization reference for the relative quantification of Glu, Glx, and GABA and expressed the outcome measure from ^1^H MRS as Glu/NAA, Glx/NAA, or GABA/NAA. The rationale for using NAA as a standard is as follows: (1) the NAA level is assumed to be the same for MDD and HVs. (2) The tissue volumes for Glu and NAA are the same in the voxel, and a partial volume correction, required when using water as a reference, is avoided. We also report results using creatine (Cr) as a normalization reference, but focus on NAA because the residual errors of NAA after relaxation correction are <1 and 20% smaller than those of Cr (see Supplementary Materials).

### Statistical analyses

Demographics for the two groups were compared by a Chi-square test for categorical values and by independent-sample *t* tests for continuous values. Between-group effects for Glu and Glx, normalized to NAA and Cr, were assessed using a univariate analysis of variance (ANOVA) with fixed factors for group and sex (due to the higher percentage of women in the MDD group), covarying for MR sequence type and age, and follow-up independent-sample *t* tests. As GABA analysis was conducted in the PROBE-J subgroup only, analysis was conducted without covarying for MR sequence type. Relationships among measures were determined by Pearson correlations. Effect sizes were calculated with Cohen’s *d*, calculated by using the mean group difference and pooled standard deviation. Two-tailed statistics were used throughout with a preset α level of significance of *P* < 0.05.

## Results

### Participants

The demographics and clinical features of the 34 physically healthy patients (MDD group) and 32 HV are described in Table 1. There was no group difference in age, but a higher percentage of women was seen in the MDD group (Chi^2^ = 3.8, *P* = 0.05). MDD patients were moderately symptomatic (MADRS total = 30.7 ± 3.5), chronically ill (current episode duration = 9.4 ± 13.3 years), and medication-free for at least 2 weeks at the time of scanning [20]. MDD patients were not required to meet formal criteria for treatment resistance.

**Table 1:** Characteristics of participating subjects.

Characteristic	MDD (*N* = 34)	HV (*N* = 32)	Statistics
Age (years)	37.2±10.7	35.1±9.6	*P* = 0.25
Female sex (%)	65%	41%	*P* = 0.05
Duration of current MDE (years)	9.4±13.3	n.a.	n.a.
Age of onset: 1st episode (years)	18.0±7.9	n.a.	n.a.
Baseline MADRS total score	30.7±3.5	n.a.	n.a.
Glx/NAA	1.3049±0.4	1.0627±0.3	*P* = 0.025, *d* = 0.63
Glutamate/NAA	0.9126±0.3	0.8128±0.2	*P* = 0.005, *d* = 0.78
GABA/NAA	0.0718±0.02	0.0866±0.02 (*n* = 12)	*P* = 0.028, *d* = 0.79
NAA/water	0.0011±0.0001	0.0010±0.0001	n.s.
Glx/Cr	1.9247±0.6	1.5845±0.4	*P* = 0.028, *d* = 0.65
Glutamate/Cr	0.8352±0.3	0.7176±0.8	*P* = 0.003, *d* = 0.85
GABA/Cr	0.0984±0.02	0.1194±0.03 (*n* = 12)	*P* = 0.02, *d* = 0.83
Cr/Water	0.00074±0.0001	0.00072±0.0001	n.s.

*MDD* major depressive disorder, *HV* healthy volunteer, *MDE* major depressive episode, *MADRS* Montgomery–Åsberg Depression Rating Scale.

### Metabolites (Table 1)

#### Glx and Glu normalized to NAA and Cr levels (Table 1)

Glx/NAA (*F*_1,60_ = 5.3, *P* = 0.025, *d* = 0.63) and Glx/Cr (*F*_1,60_ = 5.1, *P* = 0.028, *d* = 0.65) were higher in the MDD group compared with HVs. Similarly, higher Glu/NAA (*F*_1,60_ = 8.7, *P* = 0.005, *d* = 0.78) and Glu/Cr (*F*_1,60_ = 9.7, *P* = 0.003, *d* = 0.85) were observed in MDD compared with HVs.

There was no significant effect of age or MR sequence at acquisition for any of these analysis other than a significant MR sequence effect for Glu/Cr. However, group effects remained significant for Glu/NAA (*P* = 0.011) and Glx/NAA (*P* = 0.047) in an analysis restricted to the PROBE-J subsample. Similarly, Glu/Cr remained significant (*P* = 0.011) and Glx/Cr showed a trend toward significance (*P* = 0.06). This further supports the likelihood that the results were not due to differences in the MR sequence used for acquisition.

#### Sex effects

Significant sex effects were seen. For Glx/NAA, the group by sex interaction (*F*_1,60_ = 4.2, *P* = 0.045) was significant, but sex was not individually significant (*F*_1,60_ = 2.5, *P* = 0.12). For Glu/NAA, sex (*F*_1,60_ = 4.7, *P* = 0.033) was significant, while the group by sex interaction showed a trend towards (*F*_1,60_ = 3.4, *P* = 0.07) toward significance. There were no significant sex effects using Cr normalization of the outcome measures.

Due to the significant sex and interaction effects, post hoc testing was conducted using the NAA normalization reference (Table 2) and found higher Glx/NAA in male MDD patients compared with both male HV (*t*_29_ = 3.8, *P* = 0.001) and female MDD patients (*t*_32_ = 2.4, *P* = 0.024). Similarly, Glu/NAA was higher in male MDD patients compared with both male HV (*t*_14.9_ = 2.3, *P* = 0.035) and female MDD patients (*t*_32_ = 2.7, *P* = 0.01). There were no between-sex, demographic or symptom differences within the MDD group (Table 2).

**Table 2:** Sex breakdown.

	MDD (*N* = 34)		HV (*N* = 32)	
	Female (*n* = 22)	Male (*n* = 12)	Female (*n* = 13)	Male (*n* = 19)
Age	38.4 ± 11.0	35.0 ± 10.4	30.7 ± 5.1	36.9 ± 9.7
MADRS total	30.1 ± 3.4	31.9 ± 3.4	n.a.	n.a.
Duration of current MDE	10.9 ± 14.5	6.7 ± 10.6	n.a.	n.a.
Age of onset: 1st episode	18.3 ± 8.2	17.7 ± 7.8	n.a.	n.a.
Glx/NAA	1.19 ± 0.3	1.51 ± 0.4*	1.09 ± 0.3	1.05 ± 0.2
Glu/NAA	0.82 ± 0.2	1.08 ± 0.4*	0.79 ± 0.3	0.83 ± 0.2
GABA/NAA^a^	0.070 ± 0.01	0.075 ± 0.02	0.09 ± 0.03	0.09 ± 0.01

*MDD* major depressive disorder, *HV* healthy volunteer, *MDE* major depressive episode, *MADRS* Montgomery–Åsberg Depression Rating Scale.

^a^GABA/NAA subsample included 12 HV (6 female and 6 male).

*P < 0.05 compared to female MDD and HV.

#### GABA normalized to NAA and Cr (Table 1)

Because the data acquired with PROBE-P were not optimized for GABA measurement, GABA was analyzed in the PROBE-J subgroup (34 MDDs and 12 HVs) only. Significantly lower GABA was observed in MDD compared with HVs for both GABA/NAA (*F*_1_,_41_ = 5.2, *P* = 0.028, *d* = 0.79) and GABA/Cr (*F*_1_,_41_ = 5.9, *P* = 0.02, *d* = 0.83). There was no significant sex imbalance in this subsample (Chi^2^ = 0.8, *P* = 0.37), nor any significant effects of age or sex.

#### Relationship across measures

**Fig. 1:**
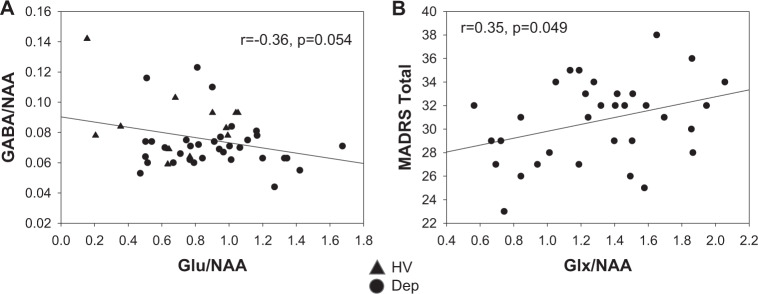
Relationships between metabolites. **A** Scatter plots of Glu/NAA vs. GABA/NAA, **B** Glx/NAA vs. MADRS total.

A trend level, an inverse correlation was present between Glu/NAA and GABA/NAA after controlling for the group (*F*_2,43_ = 3.1, *P* = 0.054, *r* = −0.36, Fig. 1A). Within-group correlations were not significant but were of a similar effect size within the MDD group alone (*r* = −0.29).

A positive correlation was present between baseline total MADRS and Glx/NAA (*r* = 0.35, *P* = 0.049, Fig. 1B). Correlations were not statistically significant between total MADRS and either Glu/NAA (*r* = 0.29, *P* = 0.1) or GABA/NAA (*r* = 0.11, *P* = 0.59). No significant correlations were seen with age or other baseline characteristics. Correlations between total MADRS and Glx/NAA were not separately significant when the groups were split by sex but were of moderate effect size and positive for females (*r* = 0.4, *P* = 0.06). Correlations using the Glx/Cr reference were not significant.

## Discussion

The principal findings in this cross-sectional study of chronically ill, not recently medicated, and symptomatic MDD were elevated Glx and Glu and lower GABA levels referenced to NAA and Cr in vmPFC/ACC compared with HVs. Higher Glx/NAA levels correlated with more severe current depression. Our findings of elevated Glx and Glu referenced to NAA and Cr in MDD are consistent with a recent meta-analysis [27], which found lower Glx in MDD compared with HV to be strongly associated with medicated MDD. By contrast, our study only examined MDD patients off medications. In the meta-analysis, 75% of the individual studies that reported elevated Glx [34–36] and 60% of the studies that reported elevated Glu [34–38] were in medication-free MDD, also consistent with our findings.

Elevated Glx and Glu may be part of the pathogenesis of MDD or a marker of a stress response because they were related to disease severity. Although we did not require formal treatment resistance for study entry, patients were highly symptomatic, and in a current major depressive episode for almost 10 years, suggesting relative treatment resistance. Other such studies of relatively treatment-resistant depressed populations have also reported higher Glu levels, including populations with BPD [39, 40] or postpartum depression [37]. Moreover, a recent meta-analysis [41] in schizophrenia parallels our findings in MDD in both this study and our previous work [20, 21]. In this schizophrenia study, elevated Glu and Glx predict elevated symptoms, and medication (antipsychotic) reduced Glx and Glu levels. If the higher Glx and Glu reflect a causal effect on the severity of psychiatric illness, this would suggest that lowering Glx or Glu level may have a therapeutic effect. That model is consistent with our recent studies, in which the antidepressant effect of NMDAR antagonists such as ketamine [20] or DCS [21] was mediated by the degree of lowering of vmPFC/ACC Glx or Glu in MDD. Similar findings have been reported for ECT [42].

While the effects of the group were significant for both, the Glu/NAA and Glx/NAA effects were greater in males with MDD, explaining a significant group by sex interaction. Despite the higher levels in males, the correlation between total MADRS and Glx was of trend level significance in females with MDD. MDD is more prevalent in females than males worldwide [43–45], with female prevalence rates nearly 2× higher in some studies [44]. Epidemiological studies do not consistently support sex differences in disease severity [43, 46, 47]. Similarly, previous studies of healthy individuals have not reported prefrontal sex differences in Glu or Glx [48–50]. The effect of sex on Glu or Glx was also not significant in the previous meta-analysis in MDD [27], but small studies in anxiety [51] and attention deficit disorder [52] have reported sex effects for Glu. It is possible that the sex effect may also be related to sex differences in glutamate receptor gene expression [53]. Future prospective studies are needed to assess the relationship of sex and disease severity in MDD.

In a secondary finding, we found pilot data indicating lower GABA levels in vmPFC/ACC in MDD, which are consistent with lower GABA in actively depressed, but not remitted MDD [28]. Although we did not find a relationship between depression severity and GABA, we did find a trend level, the inverse correlation between Glu and GABA. This inverse correlation is consistent with our hypothetical model of excitatory/inhibitory imbalance in severe depression [54–56], and argues for further studies of therapies that may restore the optimal functioning of glutamate and GABA [57, 58]. GABA findings did not appear related to sex, but this analysis was restricted by the smaller subsample size.

Strengths of this study include a relatively large sample of chronically ill, symptomatic, and recently unmedicated MDD, and the assessment of Glx, Glu, and GABA in the same subjects in a relevant brain region, vmPFC/ACC. This study also had limitations. We used two different MR sequences in the HVs, and only one was identical to the MDD. Of all experimental parameters in the MR sequences, including magnetic field strength, scanner, localization methods, gradient, and RF pulses, the only differences are the TEs and both are relatively small (e.g., TE 68 ms vs TE 80 ms). These limitations were mitigated by using partial volume and relaxation corrections for MR sequences (Supplementary Materials), and we found similar between-group findings for Glu and Glx within the PROBE-J subsample when normalized to either NAA or Cr. We used a relatively smaller sample (34 depressed subjects vs 12 HVs) for GABA measurement from J-edited spectra. The PROBE-J sequence does not have a mechanism to suppress the contributions of macromolecules to the GABA peaks and therefore, the GABA reported here should be understood/interpreted as GABA+ [59–61]. We did not correct for all potential confounds, including smoking or menstruation, due to a lack of complete data.

We used NAA as our principal reference, because it allowed us to avoid partial volume correction as required by water as a reference. NAA has been frequently used as a reference for both Glu and GABA in normal brain [62], autism [63], Rett Syndrome [64], and glutamate-related excitotoxicity in neurological illnesses [65]. While a few [66, 67] but not all [68] studies suggest that NAA altered in MDD, this limitation was minimized by the lack of between-group differences for NAA, and the confirmatory analysis using Cr as a reference. Although we used a relatively lengthy medication washout, the MDD patients were not medication naïve, and we cannot fully rule out residual medication effects. Finally, our study focused on vmPFC/ACC only. Future studies should take advantage of developing sLASER-based ^1^H MRS technology [69] allowing simultaneous measurement of multiple regions, allowing an assessment of potential network-level disturbances [70] of glutamatergic and GABA function [71].

In conclusion, elevated Glx and Glu were found in vmPFC/ACC in recently unmedicated MDD. Glx may be a marker of depression severity. While this pathology appeared to be more pronounced in male MDD, correlations with depression severity were stronger in female MDD, suggesting that sex may be an important factor in treatment studies of MDD. Elevated Glx, or perhaps Glu, may result from a GABAergic deficit resulting in glutamatergic disinhibition and should be evaluated as a stratification biomarker for clinical trials of novel glutamatergic antagonist antidepressants.

## Supplementary information

supplementary methods
