# Supplementary material for: Ventromedial prefrontal cortex/anterior cingulate cortex Glx, glutamate, and GABA levels in medication-free major depressive disorder
Source: Transl Psychiatry. 2021 Aug 5;11:419. doi: 10.1038/s41398-021-01541-1 (PMC8342485; doi:10.1038/s41398-021-01541-1)
Supplement: Supplementary file 1 — supplementary methods [file 41398_2021_1541_MOESM1_ESM.docx]

***Ventromedial prefrontal cortex/anterior cingulate cortex Glx, Glutamate and GABA Levels in Medication-free Major Depressive Disorder***

Supplementary materials: MRI and MRS data acquisition and data processing

## Data acquisition

Protocols of data acquisition, PROBE-J (34 MDD and 12 controls) and PROBE-P (20 controls), were described in detail in previous publications^1-3^. All data were acquired on a General Electric SIGNA Premier 3T MR scanner after informed consent was obtained from the subjects according to protocols approved by local institutional review board. The procedures for the scout and structural MRI images before the MRS scans were the same for both subject groups. First, three-plane scout images were acquired, followed by a high-resolution structural MRI scan in the sagittal planes; Then, high resolution structural MRI images in the oblique axial planes parallel to the AC-PC line were acquired. We prescribed the ^1^H MRS voxel based on the sagittal and axial MR images in the vmPFC with the center of the posterior side of the voxel close to the genu of the corpus callosum with dimensions 3.0 x 2.5 x 2.5 cm^3^ (**sFigure 1**). The pulse sequence of the single voxel ^1^H MRS for the depressed subjects (n = 34) and a subgroup of healthy controls (n = 12) is PROBE-J, a commercial version of the J-editing sequence with PRESS localization^4^. The sequence parameters are as follows: TR/TE = 1500/68 ms, spectral width = 5000 Hz, FID datapoints = 4096; the numbers of saved data after averaging of every eight excitations are 32 pairs of edit-on and edit-off FIDs (free induction decays) acquired in an interleaved manner and with water suppression; and 2 for water unsuppressed FIDs were also acquired. The number of channels of the phase array coil is 8. The pulse sequence for another subgroup of healthy controls (n = 20) is PROBE-P, a commercial version of the PRESS sequence^5^, with the following parameters: TR/TE = 2000/80 ms, spectral width = 2000 Hz, FID datapoints = 1024, the numbers of saved FIDs after averaging of every eight excitations are 42 and 2 for water suppressed and water unsuppressed ^1^H MRS scans, respectively. The number of channels of the phase array coil is 32.

## MRS data processing

### General

All data were subjected to the same preprocessing steps, which include optimal combination of data from coil elements, correction of frequency and phase shifts among the FIDs, averaging the FIDs to form a SNR enhanced FID for each subject, and eliminating water residue from the water suppressed data and extracting water component from the unsuppressed water data. Specific for the data acquired PROBE-J, two sets of spectra were obtained: one is a full spectrum without editing and the other is an edited spectrum from the subtraction of edit-on and edit-off spectra. In addition to conventional data processing, we specifically performed spectral quality comparison, relaxation correction and error analysis for the two datasets with different pulse sequence parameters. In particular, both datasets (TE = 68 ms and 80 ms) have good and acceptable quality in terms of baseline distortion and linewidth broadening or distortion. We did not exclude any data from any groups. Relaxation correction and error analysis are given in the following subsections.

### Spectral fitting

The measurement of Glu and Gln was performed on the water suppressed data from the full spectrum acquired by the PROBE-J and from the spectrum acquired by the PROBE-P by spectral fitting using the QUEST method^6^. The model spectra of metabolites for the fitting were obtained by MRS scan simulation using the software MARSS^7^. The set of metabolites in the fitting include glutamate (Glu), glutamine (Gln), N-acetylaspartate (NAA), N-acetylaspartylglutamate (NAAG), creatine (Cr), phosphocreatine (PCr), choline (Ch), phosphorylcholine (PCh), and myo-inositol (mI).

The measurement of GABA+ was performed on the J-edited spectrum (i.e., the difference spectrum of the edit-on and edit-off spectra from the PROBE-J scan), using GANNET^8^, a software package dedicated to the processing and quantification of J-edited spectrum. Several modifications of the software were made to facilitate our purpose and for better quality control, which include (1) adding NAA, in addition to Cr, as a reference to quantify GABA+ and (2) controlling the range of spectral shifts to avoid the “out of range” fitting. An example of the spectral fitting is given in **sFigure 1**.

### Relaxation corrections

Effects of partial volume and relaxation were corrected for metabolite quantification when using water and/or a metabolite as a reference in the form of a ratio in the main text of the paper. The following explains the procedures in some details for data corrections.

The amplitudes of metabolites signals obtained from the fitting procedures were corrected for their relaxation times using the following equation:

$A_{corr}=A_{meas}/(1-e^{-TR/T_{1}})e^{-TE/T_{2}}$ (1)

where *A_mea_*_s_ and *A_corr_* are the measured and corrected amplitudes of the metabolite, respectively, and T_1_ and T_2_ are the relaxation times of the metabolite. The tissue fractions of metabolite and water contents, as well as T_1_ and T_2_ values for metabolites and water, were obtained from literature ^9-16^ and are also listed in **sTable 1**. The quantifications of Glu, Glx, and GABA+ were performed using total NAA (tNAA = NAA + NAAG) and total creatine (tCr = creatine + phosphocreatine), respectively, as an internal reference and were expressed as ratios of these metabolites to tNAA or tCr (eg. Glu/tNAA).

**sTable 1**. Relaxation times and percentage content of water and metabolites in brain tissues.

|  | T_1_ (s) | | T_2_ (ms) | | Content (%)* | |
| --- | --- | --- | --- | --- | --- | --- |
|  | GM | WM | GM | WM | GM | WM |
| Water** | 1.60 | 0.91 | 91.6 | 69.7 | 33.1 | 27.9 |
| NAA & NAAG | 1.47 | 1.35 | 247 | 295 | 54.2 | 45.8 |
| Cr & PCr | 1.46 | 1.24 | 152 | 156 | 56.7 | 43.3 |
| Ch & PCh | 1.20 | 1.08 | 207 | 187 | 50.5 | 49.5 |
| Glu & Gln | 1.27 | 1.17 | 185 | 146 | 61.7 | 38.3 |
| GABA | 1.84 | 1.84 | 248 | 248 | 72.0 | 28.0 |
| mI | 1.36 | 1.36 | 137 | 137 | 61.0 | 39.0 |

*) Percentage concentration with total content in all tissues to be 100%; Contents of metabolites in CSF are 0.

**) T_1_, T_2_, and content of water in CSF are 4.16s, 1642 ms, and 39%, respectively.

### Errors Analyses

1. Without correction

Let the true metabolite amplitude to be A0. The signal amplitudes measured with two different TR/TE are given as follows:

$A_{meas,1}=A_{0}\left( 1-e^{-\frac{TR1}{T_{1g}}} \right)e^{-\frac{TE1}{T_{2g}}}$ $A_{meas,2}=A_{0}\left( 1-e^{-\frac{TR2}{T_{1g}}} \right)e^{-\frac{TE2}{T_{2}g}}$ (2)

where TR1/TE1 = 2000/80 and TR2/TE2 = 1500/68 ms, and T_1g_ and T_2g_ are genuine relaxation times of the metabolite.

Without relaxation correction, the relative error of measurement 1 (TR1 = 2000 ms, TE1 = 80 ms) with respect to measurement 2 (TR2 = 1500 ms, TE2 = 68 ms) are given as:

${Error}_{w.o.corr}= \frac{A_{meas,1}-A_{meas,2}}{A_{meas,2}}$ (3)

The with relaxation time correction, the error will be:

${Error}_{corr}= \frac{A_{corr,1}-A_{corr,2}}{A_{corr,2}}$ (4)

where

### $A_{corr,i}=\frac{A_{meas}}{(1-e^{-\frac{TRi}{T_{1}}})e^{-\frac{TEi}{T_{2}}}}=A_{0}\frac{(1-e^{-\frac{TRi}{T_{1g}}})e^{-\frac{TEi}{T_{2g}}}}{(1-e^{-\frac{TRi}{T_{1}}})e^{-\frac{TEi}{T_{2}}}}$ (5)

When the T1 and T2 given in sTable 1 are correct, the errors arose from relaxation will be completely corrected and errors in Eq. 4 will be zero.

However, errors will arise when the relaxation times given in sTable 1 have errors. Give an error of 5% in all relaxation times, the errors calculated for NAA and Cr in GM and WM are given in sTable 2.

**sTable 2**. Percentage relative errors of NAA and Cr in GM and WM without relaxation corrections or with relaxation corrections using relaxation times with 5% errors.

| Metabolite/Tissue | Without correction | Correction with ±10% errors in relaxation times | | | |
| --- | --- | --- | --- | --- | --- |
|  |  | Max | Min | Mean | SD |
| NAA/GM | 10.7 | 0.81 | 0.74 | 0.02 | 0.54 |
| NAA/WM | 10.6 | 0.78 | -0.72 | 0.02 | 0.05 |
| tCr/GM | 7.35 | 0.97 | -0.88 | 0.03 | 0.61 |
| tCr/WM | 5.66 | 1.01 | -0.09 | 0.03 | 0.63 |

Without relaxation correction, the relative errors for NAA are larger than those of Cr; with relaxation correction, the relative errors of NAA are smaller than Cr. In the worst-case scenario, the errors for NAA and Cr are 0.81% and 1.02%, respectively. The means errors for NAA and Cr are close to zero.

We note that standard deviations of the relaxation times reported in the literature are smaller than 5%^12^ (ranging from 1.5% to 4.1%).

### Partial volume correction

To validate tNAA as a reference, we used the unsuppressed water to verify whether the ratio of tNAA to water differ significantly between the two groups or not. This necessitated the correction of partial volume effects on the water signal, in addition to the correction of relaxation times. In single voxel proton MRS, water signal is often used as a reference to quantify the concentrations of metabolites, such as NAA, and the concentration is expressed as NAA/Water, where NAA and water are the signal amplitudes of NAA and water, respectively. This strategy is termed the relative quantification. The accuracies of this strategy are hampered by the unknown faction of CSF in the voxel, in which the water content is virtually 100% and the metabolite content is 0. This relative concentration of a metabolite is often underestimated by the existence of CSF in the voxel, an effect of the partial volume.

The effect of partial volume in proton MRS is corrected by exclusion of the water content in the cerebrospinal fluid. To this end, we need to segment the volume of the voxel into different tissue compartments and to know the fraction of water content in each compartment. We obtained the latter from the literature ^10^ and the former by voxel segmentation, using a software package imbed in GANNET ^8^ in combination with the software package of SPM v12 (https://www.fil.ion.ucl.ac.uk/spm/).

The segmented volumetric fractions of GM, WM, and CSF in the MRS voxlel for each subject are given as *f_GM_, f_WM_*, and *f_CSF_*, respectively.

The correction factor for water is:

$\begin{matrix} CF_{GM+WM}=\frac{f_{w,GM}+f_{w,WM}}{f_{w,GM}R_{w,GM}+f_{w,WM}R_{w,WM}+f_{w,CSF}R_{w,CSF}} \\ f_{w,X}=\frac{v_{X}\rho_{w,X}}{v_{GM}\rho_{w,GM}+v_{WM}\rho_{w,WM}+v_{CSF}\rho_{w,CSF}} \\ R_{w,X}=(1-e^{-TR/T_{1,w,X}})e^{-TE/T_{2,w,X}} \end{matrix}$ (6)

where *X* = {*GM, WM, CSF*}, *v_X_* is the fraction of the volume of tissue *X* with total volume to be 1, *ρ_w,X_* is the percentage water content in tissue *X*, and *R*_w,X_ is relaxation factor of water in *X*.

When the relaxation effects are not considered, the correction factor in Equation (6) is reduced to a simple form commonly seen in the literature:

$$CF_{w,GM+WM}=1-f_{w,CSF}$$

The corrected water signal is obtained from the observed signal by the following:

$$S_{w}^{corr}=S_{w}^{obs}\cdot CF_{w,GM+WM}$$

The relative concentration of a metabolite, eg, NAA, is given by

$C_{NAA}=[\frac{NAA}{W}]=\frac{S_{_{NAA}}^{obs}}{S_{w}^{obs}(1-f_{w,CSF})}$ (7)

Equation (7) is the form commonly seen in the literature.

### Relaxation and tissue volume correction for metabolites

In our relaxation correction for metabolites, we took both the volumetric fractions and relaxations of tissues of metabolites into account.

The correction factors of a metabolite in GM (or WM) and in brain tissue, respectively, are

$\begin{matrix} CF_{m,X}=1/R_{m,X}=e^{TE/T_{2,w,X}}/(1-e^{-TR/T_{1,w,X}}) \\ CF_{m,GM+WM}=\frac{f_{m,GM}}{f_{m,GM}+f_{m,WM}}CF_{m,GM}+\frac{f_{m,WM}}{f_{m,GM}+f_{m,WM}}CF_{m,WM} \\ =CF_{m} \end{matrix}$ (8)

where *f_m,X_* and *R_m,X_* are defined in Equation 2 with the subscript *w* substituted by *m* for metabolite; $CF_{m,X}$corrects for the relaxation only and $CF_{m,GM+WM}$for corrects relaxation, partial volume, and percentage content of brain tissues.

$S_{m}^{corr}=S_{m}^{obs}CF_{m}$ (9)

### Corrections of metabolite ratios

From Equation (9), the corrected ratio of two metabolite signals is given as,

$(\frac{S_{m}}{S_{rm}})^{corr}=\frac{S_{m}^{obs}}{S_{rm}^{obs}}(\frac{CF_{m}}{CF_{rm}})$ (10)

where *rm* refers to the reference metabolites, such as Cr or NAA, and the ratio of *CF_m_*/*CF_rm_* is the correction factor for the metabolite ratio of *m* and *rm*.


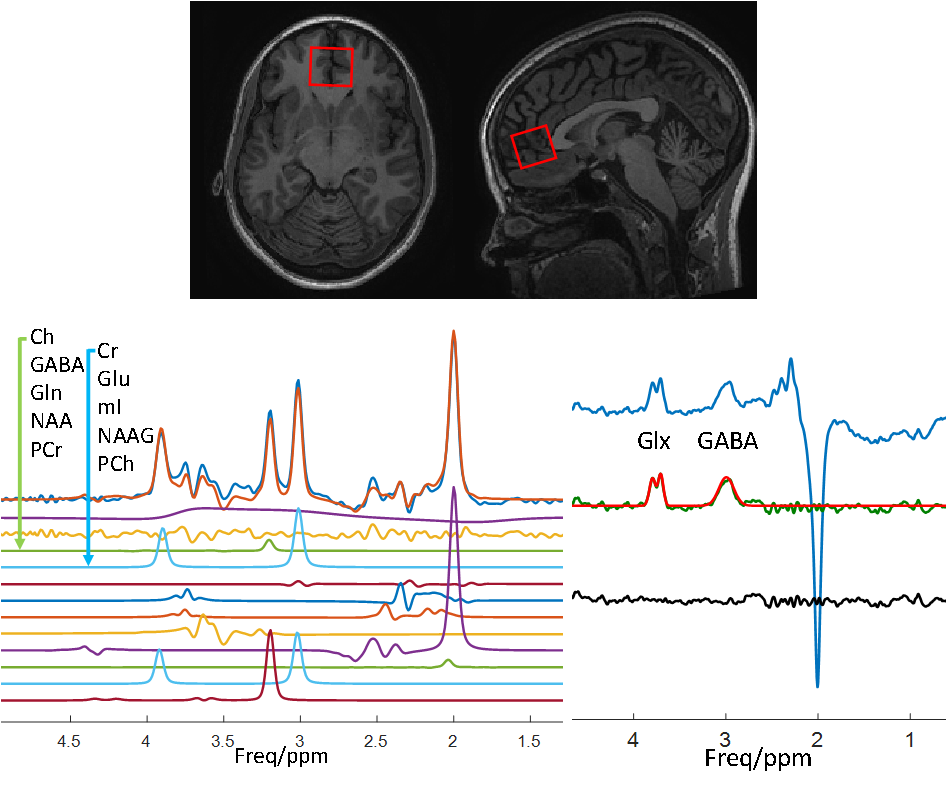


**sFigure 1**. Examples of voxel placement and ^1^H MRS spectral fitting for the PROBE-J acquisitions.

The upper panel shows locations of the ^1^H MRS voxel in the medial prefrontal cortex (vmPFC) on axial and sagittal localizer images.

The lower left panel shows spectral fitting of a full spectrum acquired with TE = 68 ms and without editing pulses. From top down to bottom are the original spectrum (blue) overlaid with the fitting spectrum (red), the background contributed from macromolecules, the residue of the fitting, and the component spectra of 10 metabolites: Ch, Cr, GABA, Glu, Gln, mI, NAA, NAAG, PCr, and PCh.

The lower right panel shows (from top to bottom) the edited ^1^H MRS spectrum, the fitting of brain GABA+ and Glx (red) overlaid on the edited GABA+ and Glx peaks (red) after other peaks including NAA at 2.0 ppm, and the residuals after peak fitting (black).

###

## Reference

1. Javitt DC, Carter CS, Krystal JH, Kantrowitz JT, Girgis RR, Kegeles LS *et al.* Utility of Imaging-Based Biomarkers for Glutamate-Targeted Drug Development in Psychotic Disorders: A Randomized Clinical Trial. *JAMA Psychiatry* 2018; **75**(1)**:** 11-19.

2. Milak MS, Rashid R, Dong Z, Kegeles LS, Grunebaum MF, Ogden RT *et al.* Assessment of Relationship of Ketamine Dose With Magnetic Resonance Spectroscopy of Glx and GABA Responses in Adults With Major Depression: A Randomized Clinical Trial. *JAMA Netw Open* 2020; **3**(8)**:** e2013211.

3. Kantrowitz JT, Milak MS, Mao X, Shungu DC, Mann JJ. d-Cycloserine, an NMDA Glutamate Receptor Glycine Site Partial Agonist, Induces Acute Increases in Brain Glutamate Plus Glutamine and GABA Comparable to Ketamine. *The American journal of psychiatry* 2016; **173**(12)**:** 1241-1242.

4. Mescher M, Merkle H, Kirsch J, Garwood M, Gruetter R. Simultaneous in vivo spectral editing and water suppression. *NMR Biomed* 1998; **11**(6)**:** 266-272.

5. Bottomley PA. Spatial localization in NMR spectroscopy in vivo. *Annals of the New York Academy of Sciences* 1987; **508:** 333-348.

6. Naressi A, Couturier C, Devos JM, Janssen M, Mangeat C, de Beer R *et al.* Java-based graphical user interface for the MRUI quantitation package. *MAGMA* 2001; **12**(2-3)**:** 141-152.

7. Landheer K, Swanberg KM, Juchem C. Magnetic resonance Spectrum simulator (MARSS), a novel software package for fast and computationally efficient basis set simulation. *NMR Biomed* 2019**:** e4129.

8. Edden RA, Puts NA, Harris AD, Barker PB, Evans CJ. Gannet: A batch-processing tool for the quantitative analysis of gamma-aminobutyric acid-edited MR spectroscopy spectra. *J Magn Reson Imaging* 2014; **40**(6)**:** 1445-1452.

9. Quadrelli S, Mountford C, Ramadan S. Hitchhiker's Guide to Voxel Segmentation for Partial Volume Correction of In Vivo Magnetic Resonance Spectroscopy. *Magn Reson Insights* 2016; **9:** 1-8.

10. Ernst T, Kreis R, Ross B. Absolute quantitation of water and metabolites in the human brain. I. Compartments and water. *J Magn Reson B* 1993; **102**(1)**:** 1-8.

11. Choi C, Coupland NJ, Bhardwaj PP, Kalra S, Casault CA, Reid K *et al.* T2 measurement and quantification of glutamate in human brain in vivo. *Magn Reson Med* 2006; **56**(5)**:** 971-977.

12. Wang Y, Li SJ. Differentiation of metabolic concentrations between gray matter and white matter of human brain by in vivo 1H magnetic resonance spectroscopy. *Magnetic resonance in medicine : official journal of the Society of Magnetic Resonance in Medicine / Society of Magnetic Resonance in Medicine* 1998; **39**(1)**:** 28-33.

13. Puts NA, Barker PB, Edden RA. Measuring the longitudinal relaxation time of GABA in vivo at 3 Tesla. *J Magn Reson Imaging* 2013; **37**(4)**:** 999-1003.

14. Traber F, Block W, Lamerichs R, Gieseke J, Schild HH. 1H metabolite relaxation times at 3.0 tesla: Measurements of T1 and T2 values in normal brain and determination of regional differences in transverse relaxation. *J Magn Reson Imaging* 2004; **19**(5)**:** 537-545.

15. Harris AD, Puts NAJ, Wijtenburg SA, Rowland LM, Mikkelsen M, Barker PB *et al.* Normalizing data from GABA-edited MEGA-PRESS implementations at 3 Tesla. *Magn Reson Imaging* 2017; **42:** 8-15.

16. Mlynarik V, Gruber S, Moser E. Proton T (1) and T (2) relaxation times of human brain metabolites at 3 Tesla. *NMR Biomed* 2001; **14**(5)**:** 325-331.
